# Bullying, social support and adolescents’ mental health: Results from a follow-up study

**DOI:** 10.1177/1403494820921666

**Published:** 2020-05-26

**Authors:** Regine Ringdal, Hanne Nissen Bjørnsen, Geir Arild Espnes, Mary-Elizabeth Bradley Eilertsen, Unni Karin Moksnes

**Affiliations:** 1 Department of Public Health and Nursing, Faculty of Medicine and Health Sciences, Norwegian University of Science and Technology, Norway; 2 NTNU Center for Health Promotion Research, Norway

**Keywords:** Mental well-being, anxiety, depression, mental health, adolescents, bullying, social support

## Abstract

**Aims:** The aim of this study was to examine the predictive roles of being bullied and perceived social support in association with adolescents’ mental health. **Methods:** At two time points, September 2016 and April–June 2017, questionnaires were distributed to students between 15 and 21 years of age in four upper-secondary schools in Norway, with a total sample size of 351. Random- and fixed-effects regression models were used to estimate the effects of being bullied and social support on adolescents’ mental health. **Results:** In the random-effects models, being bullied was associated with lower scores on mental well-being and higher scores on anxiety and depression symptoms. Social support from family and friends was associated with higher scores on mental well-being, as well as fewer anxiety and depression symptoms. However, the results from the fixed-effects model, with more realistic assumptions, indicated that being bullied was only associated with more anxiety and depression symptoms, while support from friends was associated with higher scores on mental well-being and fewer anxiety and depression symptoms. ***Conclusions*: Based on the fixed-effects models, being bullied was associated with more anxiety and depression symptoms. However, being bullied was not significantly associated with mental well-being. Social support from family was not significantly associated with either aspects of mental health. Moreover, social support from friends was associated with higher scores on mental well-being and fewer anxiety and depression symptoms. The two sources of social support did not buffer the negative effects of being bullied on either aspect of mental health.**

## Introduction

Mental health in adolescence has become an important public-health concern in recent years [1]. Symptoms of anxiety and depression are the most commonly reported mental-health problems among Norwegian adolescents [2]. Mental-health problems among adolescents have been the subject of many studies [3], while there have been fewer studies concerning mental well-being [4]. To gain more comprehensive insight on how adolescents cope with their everyday lives, it is crucial to include both positive and negative aspects of mental health [5] and examine the important role of risk and protective factors related to adolescents’ overall mental health.

Two established predictors of mental health are the experience of being bullied and perceived social support [6–9]. Many adolescents experience being bullied, and previous studies have indicated that being bullied may be associated with mental-health problems, such as anxiety and depression, in both the short and long term [5,10–12]. Several studies have found significant associations between social support and overall mental health [10,13,14]. However, there are still unanswered questions regarding the predictive roles of different sources of social support on adolescents’ overall mental health, as previous research on this field has been lacking [15]. In addition, there have been inconsistent results concerning the role of social support in the relationship between experiences of being bullied and mental-health problems [12,16].

This study is a follow-up of the study by Ringdal et al. [17] who examined the impact of social support, bullying and school-related stress in association with adolescents’ mental well-being and anxiety and depression symptoms by using cross-sectional data. Cohort data make it possible to estimate the effects of these predictors with more advanced statistical regression models which can control for stable unmeasured confounders.

Therefore, the aim of the study was to examine the predictive roles of being bullied and perceived social support from family and friends in association with adolescents’ mental health, taking advantage of statistical models for cohort data. The aim was examined through the following hypotheses:

H1: Experience of being bullied will increase anxiety and depression symptoms and decrease mental well-being.
H2: Social support from friends and family will decrease anxiety and depression symptoms and increase mental well-being, as well as buffer the negative effects of bullying on mental health.

## Method

### Participants

The study was based on longitudinal data of adolescents from four upper-secondary schools in the largest city of mid-Norway. Data were collected at two time points during the academic school year of 2016/2017 (T1: September 2016; T2: April–June 2017). Originally, five schools participated in the study. However, one of the schools withdrew after the first data collection. Therefore, only four schools were included in the statistical analysis. The four schools represent two of four districts in this city, although all districts are relatively similar in sociodemographic structure. The schools offer a wide variety of vocational and academic study tracks in both rural and urban areas and represents typical Norwegian upper-secondary schools. The number of students at each school ranged from 260 to 1087.

**Figure 1.**
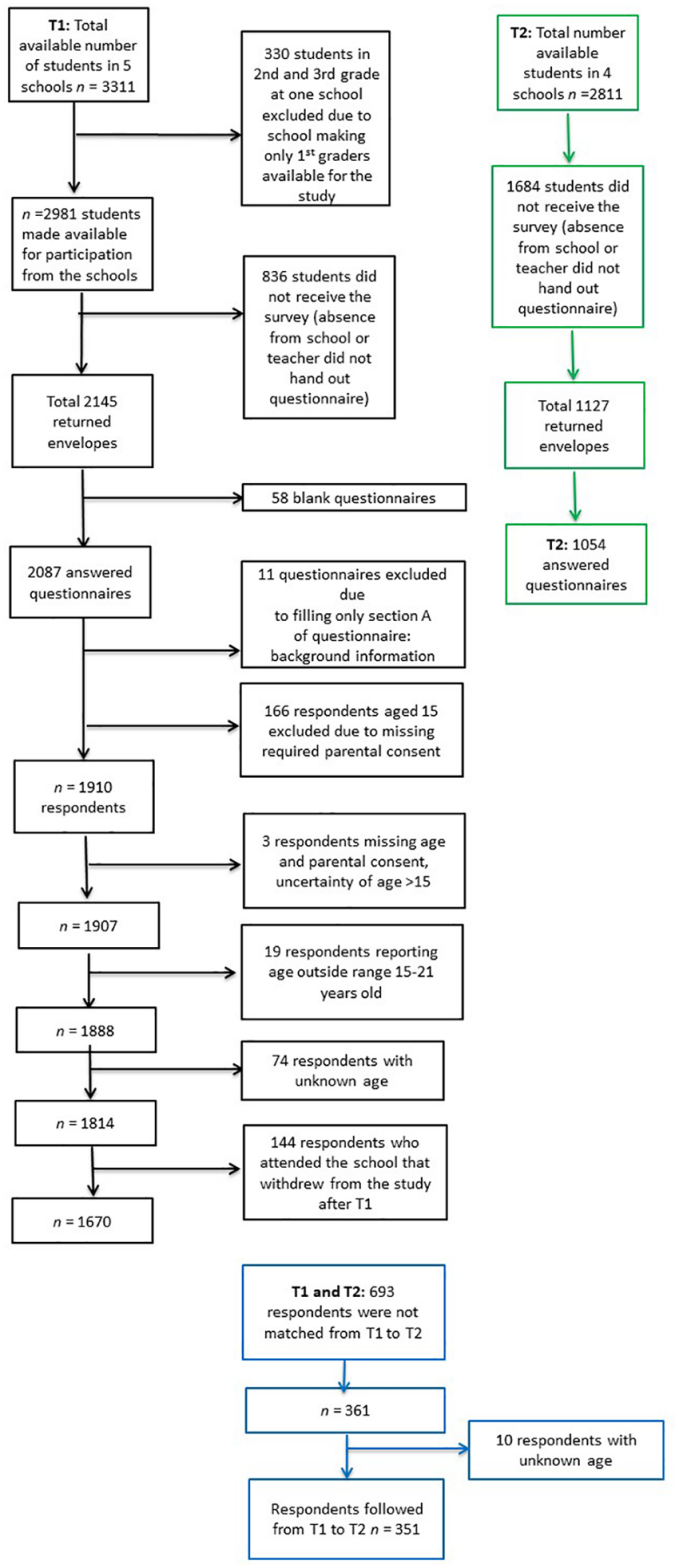
Flow chart of the participant flow from T1 to T2.

The survey administration at both time points depended on the teachers’ willingness to administer the questionnaire. The matching of students from T1 to T2 was based on seven letters taken from the responses to four questions. Many of the students did not fill out the questions needed to create the ID variable, which resulted in identical identification codes for these students. As a result, we were only able to match 361 (34.2%) students from T1 to T2. The age range was 15–21 years, and the total sample size in this study was therefore 351. The participant flow from T1 to T2 is presented in Figure 1.

### Procedure and ethics

The Regional Committee for Medical and Health Research Ethics in Central Norway (REK midt 2014/1996) approved the study. Prior to data collection, the school principals gave their informed consent for the study. Teachers were responsible for administering the questionnaires during a 45-minute classroom session of their choice. The study-specific questionnaire consisted of both validated and primary recognised scales related to school health services, mental health, family, friends, coping and experience of stress. The students were informed that participation was voluntary and anonymous through written letters, a video made by the research group available on the school’s e-learning platforms and oral information provided by teachers in each class prior to distributing the questionnaires. In accordance with §17 of the Norwegian Act on Medical and Health Research [18], all first-year students received an informational letter with a consent form to be completed by parents of students who were ⩽15 years old. Students aged ⩾16 years gave their consent by completing the questionnaire. Students who chose not to participate in the survey could do other schoolwork.

### Measures

#### Mental well-being

Mental well-being and symptoms of anxiety and depression were both used as outcome variables, assessing positive and negative aspects of mental-health, respectively. The Short Warwick-Edinburgh Mental Well-being Scale (SWEMWBS), which has been validated for adolescents, was used to measure mental well-being [3,19,20]. The adolescents were asked how they had felt about seven positively worded statements over the previous two weeks. The response scale ranged from 1=‘none of the time’ to 5=‘all of the time’, where high scale scores indicated high levels of mental well-being. In accordance with past studies [21], the SWEMWBS was estimated as a summated scale ranging from 7 to 35.

#### Anxiety and depression symptoms

Symptoms of anxiety and depression were assessed using the 10-item Hopkins Symptom Checklist, which has previously been validated for adolescents [22]. Six of the items are related to depression, while four are indicators of anxiety. The response scale ranged from 1=‘not at all’ to 4=‘extremely’, where high mean scores indicated a high level of anxiety and depression symptoms. The scale was constructed as the mean of the individual item scores, with two missing values allowed [22].

#### Bullying

The bullying scale was based on three items generated from the questionnaire for adolescents in the national Norwegian Survey on Living Conditions 2012 [23] with additionally a new item assessing cyberbullying [17]. The bullying scale was pilot tested among adolescents in one upper-secondary school in Norway (*n*=479), with a response rate of 98% in the spring of 2016. The review of the pilot led to a few minor changes in the wording of the items. The adolescents were asked how often they had experienced the following: 1=‘your peers accuse you of things you have not done or cannot help doing’, 2=‘your peers show that they do not like you, e.g., by teasing, whispering or making fun of you’, 3=‘one or more peers hit you or hurt you in other ways’ and 4=‘you are pestered by peers on social media’. The response scale included 1=‘never’, 2=‘occasionally’, 3=‘at least once a month’, 4= ‘at least once a week’ and 5=‘almost every day’. The bullying scale was constructed as the mean of the individual item scores, with one missing value allowed. High scale scores indicated more extensive experience of being bullied.

#### Social support

The Multidimensional Scale of Perceived Social Support (MSPSS) was used to measure social support [24]. The MSPSS consists of 12 items measuring perceived social support from three sources: family, friends and significant others. Previous studies have tested the psychometric properties of the scale among adolescents [25]. Only support from family and friends were included in the analyses. Both scales were constructed as the mean of the individual item scores, with one missing value allowed.

#### Background variables

Sex, age (continuous), study track, parents’ education level and perceived family income adequacy were used to describe the sample and as control variables in the regression analyses. Study track was separated in general and vocational tracks. Parents’ education level was measured by one question: ‘What is your parents’ highest level of education?’ The response scale ranged from 1*=*‘primary and lower secondary school’ to 4=‘university, more than four years’. Parents’ education level was treated as dichotomous, where 0=both parents had primary or secondary education and 1=if one or both of the parents had university-level education. Adolescents’ perceived family income adequacy was measured by one question: ‘How has your family’s income been during the past two years?’ The response scale ranged from 1=‘We have had low income the whole time’ to 5=‘We have had good income the whole time’. The variable was divided into 0=‘poor income adequacy’ and 1=‘good family income adequacy’.

### Statistical analyses

Stata v14.2 (StataCorp, College Station, TX) was used for all statistical analyses. Paired-sample *t*-tests and Pearson’s within-subject correlations for the main variables were calculated. Cronbach’s alpha was used to estimate the internal consistency (reliability) for all scales. Identical random-effects and individual fixed-effects regression models were estimated to assess the relationship between experience of being bullied, social support from family and friends and mental well-being and anxiety and depression symptoms, controlling for background variables. Hausman’s specification test was used to test the assumptions of the random-effects model [26]. The fixed-effects model controlled for stable unmeasured characteristics of the adolescents. The main drawback of the model is that the effects of time-constant variables such as sex cannot be estimated [27]. Therefore, both models were estimated. Robust standard errors were calculated to correct for effects of heteroscedasticity in all models. Missing values were deleted listwise. The significance level was set at *p*<0.05.

## Results

Table I presents a description of the sample included in this study in addition to the total sample at T1 (which only included four schools). The sex distribution and parents’ education level were rather equal in both samples. The mean age was 16.9 years at T1 and 17.5 years at T2 in this study. There were only minor differences in the distributions for perceived family income adequacy and study track for the total sample at T1 (*n*=1670) and for the total sample used in this study (*n*=351).

**Table I.** Description of the sample.

Variables	Longitudinal sample at T1, *n* (%)	Total sample at T1, *n* (%)
Sex		
Male	165 (47.1)	825 (49.7)
Female	185 (52.9)	836 (50.3)
Missing	1	9
Age (years)		
15–17	256 (72.9)	1073 (64.3)
18–21	95 (27.1)	597 (35.8)
Mean age	16.9	17.1
Study track		
Vocational	131 (37.6)	477 (29.2)
General	217 (62.4)	1157 (70.8)
Missing	3	36
Parents’ education		
Both parents with low education	133 (39.4)	642 (40.3)
One or both parents with high education	205 (60.7)	952 (59.7)
Missing	13	76
Perceived family income adequacy		
Poor	86 (24.9)	494 (30.2)
Good	259 (75.1)	1143 (69.8)
Missing	6	33
	***N*=351**	***N*=1670**

Table II presents the results from the paired-sample *t*-tests, within-subject correlations and Cronbach’s alpha. Mental well-being and anxiety and depression symptoms were the only variables with significant changes from T1 to T2. The mean sum score for mental well-being decreased significantly, while the mean score for anxiety and depression symptoms increased significantly between the two time points. All correlations within the main measures from T1 to T2 were of medium or strong sizes and were significant. The main measures showed sufficient within-subject variability for use of the fixed-effects model. The Cronbach’s alpha values were satisfactory for all scales, although the alpha for the bullying scale at T1 was slightly below 0.70.

**Table II.** Paired-sample *t*-tests, within-subject correlations and Cronbach’s alpha for the main variables.^a^

Variables	T1	T2	Mean diff. (*SD*)^b^	*t*	*r* ^c^	T1	T2
	Mean (*SD*)	Mean (*SD*)				Cronbach’s α	Cronbach’s α
Mental well-being	25.10 (5.19)	24.52 (5.35)	−0.67 (0.27)	−2.52*	0.61***	0.88	0.90
Anxiety and depression	1.72 (0.69)	1.81 (0.75)	0.09 (0.03)	3.12**	0.73***	0.92	0.93
Bullying	1.31 (0.42)	1.32 (0.53)	0.00 (0.03)	0.08	0.46***	0.67	0.81
Social support from family	4.34 (0.78)	4.34 (0.80)	0.02 (0.04)	0.62	0.66***	0.87	0.90
Social support from friends	4.37 (0.70)	4.36 (0.75)	0.01 (0.04)	0.34	0.57***	0.86	0.92

a *N*=311–332.

b Mean diff.=mean at T2 minus mean at T1.

c *r*=Pearson’s correlation between each measure at T1 and T2.

* *p*<0.05; **p<0.01; ***p<0.001.

*SD*: standard deviation.

### Regression analyses

The results from the random- and the fixed-effects regression analyses for both outcome variables are presented in Table III. An additional model for both random and fixed effects with interactions between social support and bullying was estimated for each outcome. For these models, only the results for the interactions were included in the table.

**Table III.** Summary of results from the regression analyses with mental health.

Models	Mental well-being				Anxiety and depression			
	Random effects^a^		Fixed effects		Random effects^a^		Fixed effects	
Variables	*B* ^b^	S_er_^c^	*B* ^b^	S_er_^c^	*B* ^b^	S_er_^c^	*B* ^b^	S_er_^c^
Sex (male=0, female=1)	−3.36***	0.42	–	–	0.59***	0.06	–	–
Age	−0.33	0.16	−1.03**	0.34	0.03	0.02	0.09*	0.04
Parents’ education (low=0, high=1)	0.57	0.35	–	–	0.11*	0.05	–	–
Family income adequacy (poor=0, good=1)	0.59	0.42	–	–	−0.11	0.06	–	–
Bullying	−1.48***	0.42	−0.43	0.48	0.27***	0.06	0.15*	0.06
Social support from family	1.26***	0.28	0.64	0.48	−0.09*	0.04	−0.01	0.05
Social support from friends	1.88***	0.29	1.77***	0.46	−0.17***	0.04	−0.14**	0.05
Constant	19.86***	3.27	32.70***	6.16	1.81***	0.41	0.74	0.69
*R* ^2^	0.39		0.14		0.37		0.10	
Number of observations	624		648		640		665	
Additional models^e^								
Family support×bullying	−0.73	0.53	0.02	0.71	0.08	0.08	0.01	0.08
Friends support×bullying	0.31	0.54	0.55	0.77	0.03	0.10	0.04	0.11
Hausman’s specification test	χ^2^(4)^d^=18.18**				χ^2^(4)^d^=26.27***			

a Fixed effect for school and study tracks were included in the random effects models but omitted from the table.

b *B*=unstandardized regression coefficient.

c S_er_=robust standard error.

d χ^2^=the chi-square statistic with degrees of freedom in parentheses.

e Additional models with statistical interactions. Only the results for the interaction effects are reported for these models, since the models without interaction are the best ones.

* *p*<0.05; **p<0.01; ***p<0.001.

The random-effects models included the fixed effect of schools and study tracks, controlling for stable characteristics of the school environment, although their effects are omitted from the table. However, Hausman’s specification test showed significant outcomes for both dependent variables, rejecting the assumptions of the random-effects model. Thus, we will mainly rely on the fixed-effects model for the effects of the time-varying predictors.

#### Mental well-being

The random-effects models showed that sex, bullying and support from family and friends were significantly associated with mental well-being, whereas the regression coefficients for age, parents’ education and family income adequacy were not significant. Girls scored on average 3.4 points lower than boys on the mental well-being scale. A difference of one point on the bullying scale was associated with an increase on the mental well-being scale of 1.5 points. A one-point difference on the social support scales was predicted to yield increases of 1.3–1.9 points on the mental well-being scale, with the strongest effect for social support from friends.

The fixed-effects model showed that only age and social support from friends had significant effects when controlling for all measured and unmeasured time-constant variables. Thus, one year of ageing predicted a reduction in the score on the mental well-being scale by approximately one point. An increase of one point in social support from friends predicted an average increase of 1.77 points on the mental well-being scale. The interaction effects between social support from family and friends and being bullied on mental well-being were not, however, statistically significant.

#### Anxiety and depression symptoms

The results from the random-effects model indicated that sex, parents’ education level, bullying and social support from family and friends were significantly associated with anxiety and depression symptoms. Girls scored on average 0.6 higher than the boys on the anxiety and depression scale. Adolescents with university-educated parents had slightly higher scale scores than adolescents with parents who were not educated at that level. An increase of one point on the bullying scale gave an average increase of 0.3 points on the anxiety and depression scale. A one-point difference on the social support scales was associated with decreases on the anxiety and depression scale of −0.09 for family and −0.17 for support from friends.

The fixed-effects model showed that bullying and social support from friends were the only variables with significant regression coefficients. Adolescents ageing one year experienced an increase in the anxiety and depression score of 0.09, on a scale ranging from 1 to 4. A one-point increase on the bullying scale from T1 to T2 predicted an increase of 0.15 on the anxiety and depression scale. Furthermore, an increase in social support from friends of one point predicted a decrease in the anxiety and depression scale of 0.14. The interaction effects between social support from family and friends and being bullied on anxiety and depression symptoms were not statistically significant.

## Discussion

The aim of this study was to examine the predictive roles of being bullied and perceived social support from family and friends in association with adolescents’ mental health. The discussion and conclusion on the time-varying predictors are based on the results from the fixed-effects models with more realistic assumptions than the random-effects models.

Being bullied was significantly and positively associated with anxiety and depression symptoms but was not significantly associated with mental well-being. Thus, we found only partial support for the first hypothesis. This finding is in accordance with previous studies showing both short- and long-term negative consequences of being bullied on mental health [6,10,11,28,29]. However, it should be noted that the estimated effect was small. The results indicate that although experience of bullying may lead to more anxiety and depression symptoms, it may still be possible to maintain mental well-being.

Social support from family was not significantly associated with either mental well-being or anxiety and depression symptoms. One possible explanation is that although most adolescents have close bonds to their parents and perceive them as stable sources of social support, adolescents usually become more independent from their parents, test boundaries, form closer bonds with their friends and rely more on their peers [30]. Previous research has shown inconsistent results regarding the role of family support in adolescence [10,30]. In line with our results, one study indicated that support from friends exceeded support from parents [30]. Another study indicated that parental support is a strong predictor of positive adjustment in adolescence [10].

Social support from friends was associated with higher scores on mental well-being and fewer anxiety and depression symptoms, with the strongest estimated effect for the former. These results are supported by previous studies [10,29] and underline that the quality of social relations with friends is an important coping resource which provides individuals with the feeling that they are supported and accepted and have resources available to manage challenging situations. Thus, the second hypothesis was only supported with regards to social support from friends.

Furthermore, the results showed no significant statistical interactions between the two sources of social support and being bullied on either mental well-being or anxiety and depression symptoms. Thus, the second hypothesis with regards to the buffering effect of social support was not supported. In line with our results, a recent study indicated that different sources of social support did not buffer the relationship between experiences of being bullied and mental-health problems [12]. However, social support may still be considered an important coping resource for adolescents who are being bullied [16].

The main strength of this study is the longitudinal design with measurements at two time points. This design allowed for the use of the individual fixed-effects model which controls for unmeasured stable characteristics of the adolescents. Furthermore, this study included both positive and negative aspects of mental health as outcome variables.

However, the individual fixed-effects model has some limitations. Since the estimates from the model are solely based on within-subject variation, the standard errors are larger than in the random-effects model, where the estimates also utilise the between-subject variation. The standard errors of social support from family increased from the random- to the fixed-effects model for mental well-being, which may indicate somewhat restricted variation in the variable over a school year. The within-subject variation is also vulnerable to random measurement errors, especially in situations with small changes. The observation period of one school year is short, and it is possible that the impact of being bullied was under-estimated.

Another limitation is that we were only able to match 34.2% of the students from T1 to T2. This is partly due to the teachers who served as gatekeepers of participation in the survey and partly due to the failure to match the students with the ID code. We have, however, no reason to believe that these factors involved systematic selection. Furthermore, in the fixed-effects regression model, the respondents function as their own controls and are therefore less exposed to selection bias.

## Conclusions

The results from the fixed-effects models indicated that experience of being bullied was associated with more anxiety and depression symptoms, although the effect was small. However, experience of being bullied did not seem to be significantly associated with adolescents’ mental well-being. Furthermore, social support from family was not significantly associated with the adolescents’ overall mental health. In contrast, social support from friends was associated with higher scores on mental well-being and lower scores on anxiety and depression symptoms. The two sources of social support did not, however, buffer the negative effects of being bullied on either aspect of mental health. Still, this study indicates that it is crucial to continue the work to improve the psychosocial environment in upper-secondary schools.
